# Usability and Usefulness of SMS-Based Artificial Intelligence Intervention (Mwana) on Breastfeeding Outcomes in Lagos, Nigeria: Pilot App Development Study

**DOI:** 10.2196/65157

**Published:** 2025-07-16

**Authors:** Anisha Musti

**Affiliations:** 1 Independent Researcher, 10685-B Hazelhurst Dr. #40992Houston, TX, 77043, United States

**Keywords:** breastfeed, mother, maternal, mHealth, mobile health, mobile intervention, femtech, child mortality, maternal healthcare, pediatric, infant, newborn, artificial intelligence, SMS, text message, Nigeria, Africa

## Abstract

**Background:**

Nigeria has one of the highest child mortality rates globally, with 111 deaths per 1000 live births. Exclusive breastfeeding (EBF) improves infant survival by providing essential nutrients and antibodies that protect against infections and diseases. Despite its benefits, EBF rates in Nigeria remain low at 29%, largely due to limited health care support and breastfeeding guidance. With the proliferation of mobile phones in Nigeria, mobile health (mHealth) interventions are being explored as scalable solutions. SMS text messaging interventions have demonstrated success in delivering behavioral interventions; yet, few use artificial intelligence (AI) for personalized breastfeeding support.

**Objective:**

This study evaluates the effectiveness of Mwana, an AI-powered SMS-based app, in improving breastfeeding outcomes for postpartum mothers in Lagos, Nigeria.

**Methods:**

Mwana was developed using TextIt for SMS integration and Meta’s Wit.ai for natural language processing (NLP). The chatbot provides breastfeeding support via SMS, offering personalized tips, addressing common challenges, and connecting users to human agents when necessary. The intervention was piloted with 216 postpartum mothers recruited through local health care networks, focusing on usability, usefulness, and engagement. The study used a mixed methods approach, using structured surveys and observation to assess participant experiences at multiple intervals over a 6-month period. Primary outcomes measured were app usability, usefulness, and breastfeeding adherence.

**Results:**

The intervention was well-received, with high scores for both usefulness (mean 4.01, SD 1.41) and usability (mean 3.92, SD 1.35) on a 5-point scale. The majority of respondents, 57% (118/206), rated the chatbot’s usefulness at the highest score of 5. Qualitative feedback statements identified areas for improvement, including enhancing AI comprehension, response times, and human-like interaction.

**Conclusions:**

The study highlights the potential of Mwana to improve breastfeeding outcomes in resource-limited settings, contributing to the growing body of evidence supporting mHealth interventions in maternal and child health. By leveraging personalized messaging, SMS-based delivery, and language localization, Mwana offers a scalable, accessible solution. However, challenges remain regarding AI comprehension, and further research is necessary to evaluate Mwana’s effectiveness among populations not actively engaged with health care services. Future iterations will expand AI training datasets, refine NLP capabilities, and scale to broader populations.

## Introduction

### Background

In Nigeria, child mortality remains a critical public health concern. As of 2020, the under-five mortality rate was estimated at 111 deaths per 1000 live births, considering both perinatal and preterm births [1]. This rate places Nigeria as the country with the second-highest under-five mortality rate globally and the highest number of annual under-five deaths worldwide, with 844,321 deaths recorded in 2020 [2].

Breastfeeding is widely recognized as a critical intervention to improve child survival outcomes for both perinatal and preterm babies, a leading cause of child mortality [2]. Secretory immunoglobulin, a key component of breast milk, provides essential immune protection to infants, particularly against respiratory and gastrointestinal infections—two leading causes of child morbidity and mortality [3,4]. Exclusive breastfeeding (EBF) during the first 6 months of life is strongly recommended by the World Health Organization (WHO) and United Nations International Children's Emergency Fund (UNICEF) to optimize infant health outcomes [5]. Despite its benefits, Nigeria’s EBF rate remains low at approximately 29%, far below the global target of 70% by 2030 [6]. Addressing this gap could prevent over 95,000 child deaths annually in Nigeria [7].

One of the key barriers to improving EBF rates in Nigeria is the lack of adequate support within the health care system. Many mothers struggle to access accurate and timely information on breastfeeding techniques, proper positioning, and other challenges. Furthermore, limited counseling from health care providers often results in suboptimal breastfeeding practices [8]. While breastfeeding is widely practiced in Nigeria, the persistently low EBF rate of 29% highlights the gap between awareness and sustained EBF [9].

### Prior Work

The rapid growth of mobile phone use has significantly impacted health care delivery, particularly in resource-limited settings [10]. With increasing accessibility and affordability, mobile technology—especially SMS—offers a powerful tool for delivering targeted health interventions to mothers [11]. Mobile health (mHealth) initiatives have already demonstrated success in improving maternal and child health outcomes [11].

In Nigeria, where mobile phone penetration continues to rise, mHealth tools could help address barriers to EBF by providing mothers with timely, accessible information [12]. With approximately 88% of women owning basic cell phones [13], mobile technology presents a promising avenue for intervention. However, research on integrating artificial intelligence (AI) into SMS-based breastfeeding interventions remains limited.

One example of mobile technology leveraging AI is the iCow app. Developed by Su Kahumbu, iCow was originally created to be a mobile-based calendar for farmers in Tanzania and Ethiopia. With nearly 60,000 registered users, iCow illustrates the potential of mobile apps to effectively engage large populations, a relevant insight for Nigeria’s expanding smartphone user base [14].

### Su Kahumbu

Su Kahumbu originally developed iCow as a mobile-based calendar for farmers in Tanzania and Ethiopia. With nearly 60,000 registered users, iCow illustrates the potential of mobile apps to effectively engage large populations—a relevant insight for Nigeria’s expanding smartphone user base [14].

Similarly, the Moommae mobile app, developed by P Chaovalit and S Pongnumkul, was studied as a breastfeeding intervention in Thailand. It offered tracking tools for feeding activities and support for breastfeeding outside the home. While assessments confirmed its potential as a self-management tool, usability challenges underscored the need for refinements to enhance the user experience [15].

These findings suggest that mobile-based interventions can positively influence breastfeeding behaviors. However, further research is needed to explore AI-driven SMS apps for improving breastfeeding support in Nigeria.

### Goals of This Study

This research study aims to evaluate the effectiveness of an SMS-based AI app in improving breastfeeding outcomes for mothers in Lagos, Nigeria. The intervention leverages culturally accepted breastfeeding practices in Nigeria while addressing gaps in medical support through an AI-powered virtual assistant. Specifically, we implemented Mwana using TextIt and Meta AI, piloted it with a focus group of postpartum mothers in Lagos, Nigeria, and evaluated our implementation using usability and usefulness metrics to assess its potential for long-term impact on breastfeeding practices.

By focusing on EBF as a key strategy for reducing child mortality, this research contributes valuable insights into the role of AI-driven mHealth interventions in resource-limited settings.

## Methods

### Study Design

This formative pilot study combined extensive opinion surveys and observational data collection to assess the feasibility and effectiveness of the SMS-based AI intervention, Mwana, in supporting breastfeeding mothers.

Data collection involved externally administered surveys that captured both quantitative engagement metrics and qualitative participant experiences. The surveys included structured response options to track breastfeeding adherence, response rates, and interaction patterns with the intervention, alongside open-ended questions that allowed mothers to share insights on their challenges, preferences, and overall experience using Mwana. Multiple data collection points were scheduled at specific intervals, including at the commencement of the study, at 1 month, 3 months, and 6 months later. No formal interim analyses were conducted due to the small sample size and exploratory nature of the pilot study.

In addition, an observational pilot study was conducted to evaluate how Mwana was integrated into postpartum care without direct intervention or variable manipulation. Platform use was remotely monitored and recorded, focusing on its role in breastfeeding routines among postpartum mothers. The study spanned 6 months (January 1, 2023, to July 31, 2023) in Lagos, Nigeria, with all participant interactions occurring through the platform and surveys, without any in-person engagement.

No major methodological changes were made after the trial commenced. However, based on preliminary user feedback in the first month, additional predefined responses were introduced to improve the chatbot’s comprehension of common breastfeeding concerns, and message queuing was optimized to reduce response delays.

### Participants and Recruitment

Participants were recruited using convenience sampling. Postpartum mothers were recruited from local health care networks, including Breastfeeding Support Lagos, the Family Youth and Health Initiative, and the Ibadan North East Primary Health Care Center in Oyo. Local partners identified and contacted eligible participants during routine antenatal and postnatal visits, provided study details, and obtained informed consent prior to enrollment (Textbox 1).

Textbox 1. **Eligibility criteria for the Mwana pilot.Inclusion criteria**

- Within 9 months post partum.
- Owns a mobile phone with SMS capability.
- Provides consent to engage with Mwana for 6 months.
- Access to the internet is required to complete the survey questionnaires.

Exclusion criteria

- Mothers without access to an SMS-capable mobile phone, as the intervention relied on text-based engagement.
- Unwilling to participate for the full 6-month period.
- Mothers with medical conditions significantly affecting breastfeeding were excluded to ensure data consistency.

The sample size of 216 mothers was determined based on feasibility and the need for a sufficiently large cohort to capture diverse breastfeeding behaviors and engagement patterns. This number ensured adequate representation across various maternal health care settings while maintaining logistical feasibility for data collection and follow-ups. Efforts were made to ensure a diverse and representative sample, including mothers from both urban and periurban areas. Participants were randomly selected based on breastfeeding status to maintain a balanced representation of exclusive and non-EBF mothers.

Among the enrolled mothers, 85.6% had been pregnant within 6 months of participation, with all within 9 months. Most had prior access to maternal health care, with 88 attending specialist hospitals, 46 visiting general hospitals, and 72 using other health care facilities.

### Software and Technology

#### Operating Mode of the Mwana Platform

The Mwana platform was designed as an SMS-based intervention to provide breastfeeding support, eliminating the need for a mobile app. SMS was chosen due to its broad accessibility and lower technological barriers compared to smartphone apps [16]. While smartphone penetration is increasing, many individuals—particularly in low-resource settings—still rely on basic feature phones with limited internet connectivity [17]. By leveraging SMS, Mwana ensured that the intervention was available to a wider audience, making it a cost-effective and scalable solution.

In addition, SMS is a widely used and familiar communication method in Nigeria, increasing the likelihood of participant engagement [18]. Unlike mobile apps that require regular updates and compatibility across operating systems, SMS-based solutions require minimal infrastructure while maintaining high usability and accessibility. As shown in Table 1, the majority of mothers preferred SMS as their communication channel (n=206), followed by WhatsApp (n=161).

**Table 1.** Distribution of mothers’ access to different communication platforms.

Category	Mother
Smart apps	74
WhatsApp	161
SMS text messaging	206

#### Language and Localization

To enhance accessibility, the Mwana platform was translated into Hausa and Swahili, two of the most widely spoken languages in the region. Hausa is commonly spoken across Northern Nigeria and West Africa, while Swahili is prevalent in East Africa, including among communities within Nigeria [19].

To ensure linguistic accuracy and cultural appropriateness, the translation process was conducted in collaboration with local translators who had in-depth knowledge of both language nuances and cultural contexts. This approach ensured that all messages, prompts, and interactions were not only grammatically correct but also culturally sensitive and relevant, ultimately improving user engagement and comprehension.

#### Mwana System Architecture

The Mwana system architecture is designed to provide structured, AI-powered maternal health support through an interactive chatbot interface. It integrates TextIt for building conversation flows and Wit.ai for backend natural language processing (NLP), ensuring smooth and dynamic interactions with users.

The system begins processing when a user submits a query, which the AI interprets as an utterance—a user-provided phrase such as “I have nipple pain.” These utterances are analyzed using a training dataset of 7000 prior utterances to identify the intent behind the query. Wit.ai, a machine learning–based NLP engine, performs this classification, mapping the user’s statement to a predefined intent such as “breastfeeding pain” or “latching issues.”

Once the AI successfully determines the intent, the system routes the user to the appropriate predesigned flow within TextIt. TextIt structures interactions using a decision-tree format, where users navigate a sequence of questions and responses. Each flow consists of a series of conditional prompts—typically yes or no questions—that guide users toward a resolution. For example, if a user reports nipple pain, the system may ask follow-up questions such as “Is the pain sharp or dull?” and “Does the pain persist after feeding?” to refine the diagnosis and provide relevant guidance.

By leveraging Wit.ai for intent recognition and TextIt for structured decision-tree flows, Mwana delivers a responsive and scalable maternal support system, ensuring that users receive context-aware assistance tailored to their specific concerns.

#### Mwana User Experience Design and Functionality

**Figure 1.**
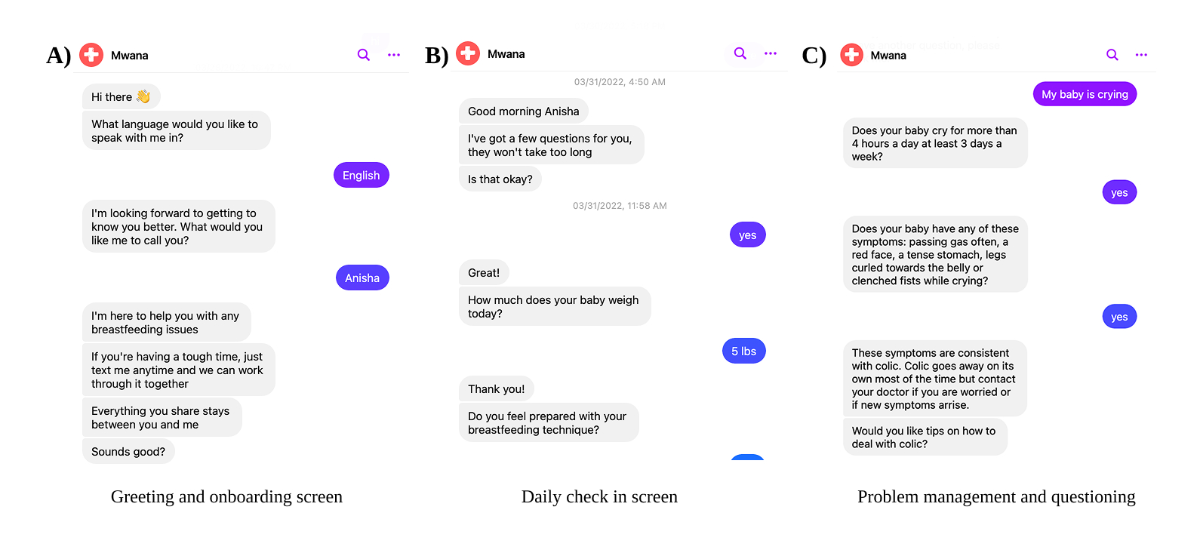
Selection of Mwana chatbot interface screenshots, illustrating different user interactions. (**A**) Greeting and onboarding screen: the chatbot introduces itself, asks for the user’s preferred language and name, and reassures the user about privacy and support for breastfeeding issues. (**B**) Daily check-in screen: the chatbot engages in a routine check-in, asking about the baby’s weight and the user’s confidence in their breastfeeding technique. (**C**) Problem management and questioning screen: the chatbot assesses a concern regarding a crying baby by asking follow-up diagnostic questions to determine if the symptoms align with colic, ultimately providing reassurance and guidance.

Mwana’s user design is structured to provide an intuitive and supportive experience for new mothers, ensuring seamless interaction with the AI-powered system. The interface is designed with a conversational approach, mimicking human interaction to foster engagement and trust. The chatbot’s structured flow categorizes user interactions into 3 key components: onboarding, daily check-ins, and problem management, allowing for a progressive and context-aware experience (Figure 1).

The onboarding process introduces the user to the platform, allowing personalization through language selection and name input. This personalization helps create a sense of familiarity and user-centric engagement. Daily check-ins serve as proactive touchpoints where the system collects essential health data, such as infant weight and breastfeeding confidence, reinforcing habitual engagement and real-time support. If an infant’s reported weight deviates from expected growth patterns or if a mother expresses low confidence, frustration, or distress, the system flags these as potential concerns and will initiate a predefined response system for the identified issues.

The problem management system leveraged predefined flows structured as decision trees to enable the chatbot to ask targeted questions based on the user’s concern. When an issue was identified, the platform guided users through automated support flows and, when necessary, escalated cases to human agents for further assistance. By integrating automated classification and escalation mechanisms, Mwana ensured timely and effective support while minimizing the need for continuous human intervention. This approach improved user experience, engagement, and accessibility, making breastfeeding support more efficient and responsive for postpartum mothers.

### Ethical Considerations

This study adheres to ethical guidelines for human participants research and complies with relevant regulatory standards. It is deemed exempt from Institutional Review Board review under 45 CFR 46.104(d)(2), as it involved anonymous survey data with minimal risk to participants. In addition, the research was conducted in accordance with the Declaration of Helsinki, ensuring ethical integrity and participant protection. To safeguard privacy and confidentiality, all survey data were collected anonymously and focused solely on opinions, preventing the identification of individual participants. No compensation was provided to participants, and no identifiable individual data were included in the study. Because the survey did not collect identifiable personal information and was limited to nonsensitive opinion-based questions, a formal IRB assessment was not pursued.

## Results

### Summary of User Feedback to the Mwana Platform

The feedback collected from participants in the Mwana pilot study provided valuable insights into the app’s usability, usefulness, and areas for improvement. A total of 52 feedback statements were analyzed, revealing several recurring themes that shaped user experience and satisfaction. Feedback was categorized as positive, negative (eg, delayed responses, chatbot rigidity, or comprehension issues), or mixed (Table 2).

**Table 2.** Qualitative feedback categorization from postpartum mothers using the Mwana chatbot (n=52).

Feedback categorization	Percentage of sample (%)
Positive feedback	60
Negative feedback	
Delayed, no doctor response	6
Chat rigidity, comprehension issues	12
Other	12
Mixed feedback	10

Among participants who provided written qualitative feedback, the majority (31 participants) shared positive feedback, reflecting overall satisfaction with the app. Negative feedback (16 participants) primarily centered on concerns such as delays or no doctor response (3 participants), chatbot rigidity and comprehension issues (6 participants), and other unspecified issues (6 participants). A small subset (5 participants) provided mixed feedback, indicating neither strong positive nor negative feedback. These insights underscore the platform’s strengths while also identifying key areas for improvement, particularly in chatbot responsiveness and AI comprehension.

### Statistical Analysis of Usability and Usefulness

To assess the impact of the Mwana intervention, a statistical analysis was conducted comparing baseline and postintervention scores for usability and usefulness. The results indicate a statistically significant improvement in both categories, demonstrating the intervention’s effectiveness.

Over a 6-month period, 206 of the 216 recruited postpartum mothers completed the pilot study, resulting in a 95% completion rate. Participant engagement with Mwana was measured using the average daily check-in response rate. Initially, engagement was strong, with a 90% response rate in the early weeks of the study. However, this rate gradually declined and stabilized at 60% by the end of the study period.

Analysis of engagement trends revealed that the most frequently accessed chatbot flows included “Too Much Milk,” “Breastfeeding Technique,” and “Nipple Pain,” highlighting the key areas where mothers sought support. This suggests that users primarily relied on Mwana for immediate breastfeeding guidance.

In the final evaluation, participants rated Mwana’s usability and usefulness on a 5-point scale. The majority assigned the highest rating of 5 in both categories, with 124 respondents for usefulness and 103 respondents for usability. The average usefulness score was 4.01 (SD 1.41), while usability received a slightly lower mean score of 3.92 (SD 1.35; Table 3). Notably, no participants reported injuries or safety concerns, and qualitative feedback was overwhelmingly positive, with many expressing enthusiasm for continued engagement and additional support.

**Table 3.** Distribution of usability and usefulness scores among postpartum mothers in Lagos, Nigeria (N=206).

Score	Usability response, n (%)	Usefulness response, n (%)
1	19 (9.2)	20 (9.7)
2	20 (9.7)	21 (10)
3	23 (11)	20 (9.7)
4	41 (20)	27 (13.1)
5	103 (50)	118 (57.2)

The mean usefulness score was 4.01, with a 95% CI of 3.82 to 4.20, and the mean usability score was 3.92, with a 95% CI of 3.74 to 4.10. These CIs indicate that, if the study were repeated, the true population mean would likely fall within these ranges, reinforcing the high level of user satisfaction with the intervention.

The paired *t* tests showed significant improvements in both usability and usefulness (*P*<.001), indicating that the likelihood of these results occurring by chance is extremely low. This supports the conclusion that the chatbot intervention had a real, measurable positive effect on how postpartum mothers perceived its usefulness and ease of use.

The effect size, measured using Cohen *d*, further supports the impact of the intervention. The calculated effect sizes were 0.48 for usefulness and 0.51 for usability, both indicating a moderate effect. In practical terms, this suggests that the Mwana chatbot made a meaningful difference in users’ perceptions, improving their experience in a way that was both statistically and practically significant.

## Discussion

### Principal Findings

This study assessed the feasibility and effectiveness of Mwana, an AI-driven SMS-based intervention designed to support EBF among postpartum mothers in Lagos, Nigeria. On a scale of 5, the findings indicate that Mwana was well-received, with high usefulness (mean 4.01, SD 1.41) and usability (mean 3.92, SD 1.35) scores. More than half of the participants (57%, 118/206) rated the chatbot’s usefulness at the highest level, suggesting strong demand for mobile-based breastfeeding support.

The consistently high usability and usefulness scores across the 6-month study period demonstrate the potential of SMS-based AI interventions to serve as accessible tools for maternal health support. Statistically significant improvements in both metrics (*P*<.001), along with moderate effect sizes (Cohen *d*=0.48 for usefulness and 0.51 for usability), provide compelling evidence of impact. These findings align with previous research showing that mHealth interventions can effectively support health care delivery in areas with limited resources [10,11].

A notable strength of the Mwana platform was its language localization capabilities, which enabled interactions in Hausa and Swahili alongside English. This multilingual approach expanded accessibility to diverse linguistic communities within Nigeria, addressing a critical barrier to health information delivery in multilingual settings [19]. In addition, the selection of SMS as the delivery method proved advantageous, as evidenced by the high study completion rate (95%), confirming its appropriateness in contexts with limited smartphone penetration.

Despite these positive reception indicators, the study also revealed critical challenges. The AI system struggled to accurately interpret complex user queries, leading to frustration and delayed responses. Many users reported difficulty in receiving immediate answers to their breastfeeding concerns, with some noting that they had to repeat queries multiple times before receiving a relevant response. In addition, the translation feature improved accessibility for Hausa- and Swahili-speaking mothers, but some cultural nuances in breastfeeding practices were not fully accounted for in chatbot responses.

Another key finding was engagement trends, as measured through response rates and retention over the 6-month period. While initial engagement was high, a gradual decline in response rates was observed in later months (50% reduction), suggesting that long-term user retention remains a challenge for SMS-based interventions. This could be due to the static nature of SMS interactions or the absence of multimedia content, which is known to enhance engagement in digital health interventions [20]. The most frequently accessed chatbot flows—“Too Much Milk,” “Breastfeeding Technique,” and “Nipple Pain”—provide valuable insights into the primary concerns of new mothers and can inform future content development for breastfeeding support interventions.

### Comparison With Prior Work

This study contributes to the expanding field of AI-driven mHealth interventions by introducing an SMS-based chatbot designed to support breastfeeding. Although previous mHealth solutions have shown promise in improving maternal health outcomes, few have leveraged AI to provide real-time breastfeeding guidance in low-resource environments.

Several breastfeeding support interventions have focused on mobile apps rather than SMS. For example, the Moommae app in Thailand successfully enhanced self-management behaviors among breastfeeding mothers, but its reliance on smartphone technology limited its accessibility to low-income populations [15]. Similarly, the LactApp in Spain, developed by Maria Berruezo, Alba Padró, and Enric Pallarés, offered AI-driven breastfeeding support, but its requirement for internet access restricted its use in areas with poor connectivity. In contrast, our intervention operates via SMS, ensuring that mothers with basic mobile phones and no internet access can also benefit [21].

Our findings also compare favorably with traditional SMS-based health interventions that lack AI capabilities. While conventional SMS programs typically deliver static, scheduled messages, Mwana’s interactive approach enables more personalized support based on individual user queries. This represents an important evolution in mHealth design, moving beyond one-way communication toward conversational engagement that can adapt to diverse user needs.

Other mHealth initiatives, such as iCow in East Africa, have effectively used AI-driven mobile engagement for agricultural education. While iCow demonstrated that AI can drive behavioral change through mobile messaging, its model has not been widely applied to maternal health [14]. Our study builds upon this concept by using conversational AI for real-time maternal support, marking a new era of AI-powered SMS in public health.

The observed engagement patterns in our study—high initial participation followed by gradual decline—mirror trends seen in other digital health interventions [20]. However, our retention rates remained relatively strong compared to similar studies, possibly due to the immediate practical value of the breastfeeding support provided. This suggests that when mHealth interventions address concrete, immediate needs rather than abstract health goals, they may sustain user interest more effectively.

### Limitations

This study has several limitations that should be considered when interpreting the results. First, the intervention relied solely on SMS, which limited the ability to deliver rich educational content, such as images or videos, that might enhance user understanding. This could potentially impact the overall educational impact of the intervention. In addition, while SMS-based interventions are highly accessible, participants may become desensitized to frequent messages, leading to decreased responsiveness [20].

Second, the study population was drawn from health care–engaged mothers who were already accessing maternal health services. This may limit the generalizability of findings to more vulnerable populations, such as mothers in rural areas who may not regularly visit health care facilities. Future research should explore how Mwana performs in non–health care engaged populations to assess its broader impact.

Another limitation was the chatbot’s dependency on a predefined NLP dataset. Despite training the AI model on 7000 breastfeeding-related queries, gaps in language processing remained, particularly for complex or nonstandard user inputs. Expanding the chatbot’s training dataset and refining NLP algorithms through machine learning could improve response accuracy and overall user experience.

Finally, the study faced occasional challenges with translator availability for Hausa and Swahili. Although the AI system provided automatic responses in these languages, user feedback indicated that some responses lacked cultural nuance, affecting trust in the chatbot’s guidance. Addressing this issue will require additional cultural adaptation and expert verification of translated content.

### Conclusions

This study demonstrates the feasibility of an AI-powered SMS intervention for breastfeeding support in Nigeria. Our findings indicate high usability and usefulness scores (mean 4.01, SD 1.41), aligning with our hypothesis that an AI-driven SMS intervention could serve as a feasible maternal health tool.

The intervention’s strengths—including its accessibility through basic mobile phones, multilingual capabilities, and ability to provide personalized guidance—position it as a potentially valuable complement to traditional health care services in resource-limited settings. The integration of AI with SMS technology represents an innovative approach that balances technological sophistication with accessibility. By leveraging NLP capabilities within a widely available communication channel, Mwana demonstrates a pragmatic solution that doesn’t require smartphone ownership or internet connectivity—key barriers that have limited the reach of previous mobile app interventions [15,21]. This approach has particular relevance for Nigeria, where basic mobile phone penetration is high (88% among women), but smartphone and internet access remain limited [13].

Future work should focus on expanding the chatbot’s training dataset to improve response accuracy, integrating real-time expert consultation to supplement AI-driven guidance, and testing engagement strategies to enhance long-term retention. In addition, further research should assess the impact of Mwana among nonhealth care–engaged populations to evaluate its scalability beyond the current study setting.

From a public health perspective, these findings have significant implications for addressing Nigeria’s low EBF rates. With child mortality rates remaining alarmingly high (111 deaths per 1000 live births) [1] and EBF rates at only 29% [9], scalable interventions that can effectively support breastfeeding mothers are urgently needed. Longitudinal studies measuring the impact of AI-powered SMS interventions on actual breastfeeding rates and duration would provide more definitive evidence of their public health value.

The statistically significant improvements observed in usability and usefulness, coupled with positive qualitative feedback, suggest that Mwana effectively addresses a critical gap in breastfeeding support services. Mwana represents a scalable, cost-effective solution for addressing breastfeeding support gaps in resource-limited settings. With continued development, AI-powered mHealth interventions such as Mwana have the potential to significantly improve maternal and child health outcomes in Nigeria and beyond.

## Abbreviations

AI: artificial intelligence
EBF: exclusive breastfeeding
mHealth: mobile health
NLP: natural language processing
UNICEF: United Nations International Children's Emergency Fund
WHO: World Health Organization
